# Supplementary material for: Inflammatory factors and risk of lung adenocarcinoma: a Mendelian randomization study mediated by blood metabolites
Source: Front Endocrinol (Lausanne). 2024 Aug 27;15:1446863. doi: 10.3389/fendo.2024.1446863 (PMC11384989; doi:10.3389/fendo.2024.1446863)
Supplement: Supplementary file 1 [file DataSheet1.zip › supplementary files/S2/GCST90200389/sensitivity-analysis.pdf]

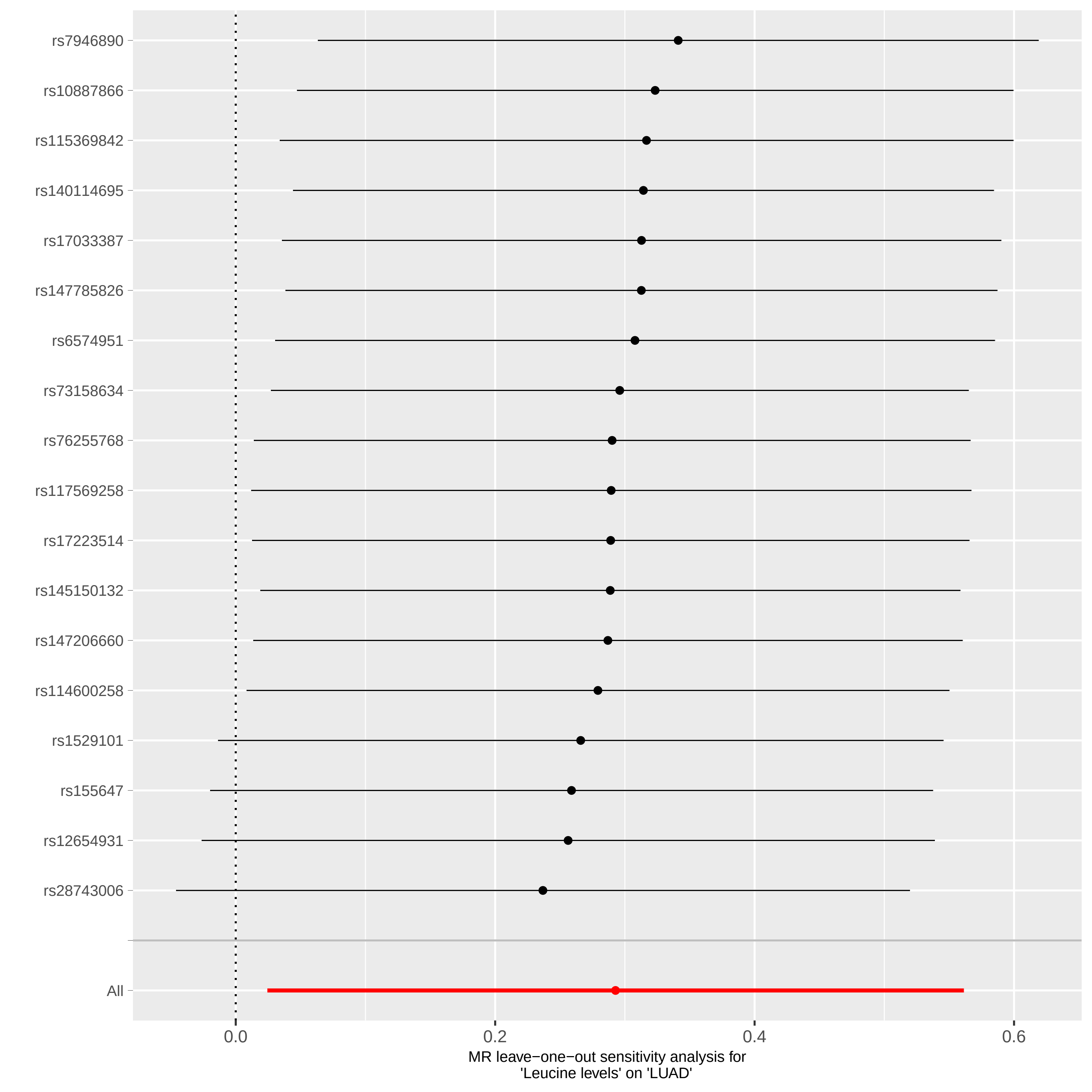

rs7946890
rs10887866
rs115369842
rs140114695
rs17033387
rs147785826
rs6574951
rs73158634
rs76255768
rs117569258
rs17223514
rs145150132
rs147206660
rs114600258
rs1529101
rs155647
rs12654931
rs28743006
All
0.0
0.2
0.4
0.6
MR leave−one−out sensitivity analysis for
'Leucine levels' on 'LUAD'
